# Distribution of the DNA transposon family, *Pokey* in the *Daphnia pulex* species complex

**DOI:** 10.1186/s13100-016-0067-7

**Published:** 2016-06-17

**Authors:** Shannon H. C. Eagle, Teresa J. Crease

**Affiliations:** Department of Integrative Biology, University of Guelph, Guelph, ON N1G 2 W1 Canada

**Keywords:** *Daphnia*, Transposon, *Pokey*, Ribosomal DNA, MITE

## Abstract

**Background:**

The *Pokey* family of DNA transposons consists of two putatively autonomous groups, *Pokey*A and *Pokey*B, and two groups of Miniature Inverted-repeat Transposable Elements (MITEs), m*Pok*1 and m*Pok*2. This TE family is unusual as it inserts into a specific site in ribosomal (r)DNA, as well as other locations in *Daphnia* genomes. The goals of this study were to determine the distribution of the *Pokey* family in lineages of the *Daphnia pulex* species complex, and to test the hypothesis that unusally high *Pokey*A number in some isolates of *Daphnia pulicaria* is the result of recent transposition. To do this, we estimated the haploid number of *Pokey,* m*Pok,* and rRNA genes in 45 isolates from five *Daphnia* lineages using quantitative PCR. We also cloned and sequenced partial copies of *Pokey*A from four isolates of *D. pulicaria*.

**Results:**

Haploid *Pokey*A and *Pokey*B number is generally less than 20 and tends to be higher outside rDNA in four lineages. Conversely, the number of both groups is much higher outside rDNA (~120) in *D. arenata*, and *Pokey*B is also somewhat higher inside rDNA. m*Pok*1 was only detected in *D. arenata.* m*Pok*2 occurs both outside (~30) and inside rDNA (~6) in *D. arenata*, but was rare (≤2) outside rDNA in the other four lineages. There is no correlation between *Pokey* and rRNA gene number (mean = 240 across lineages) in any lineage. Variation among cloned partial *Pokey*A sequences is significantly higher in isolates with high number compared to isolates with an average number.

**Conclusions:**

The high *Pokey* number outside rDNA in *D. arenata* and inside rDNA in some *D. pulicaria* isolates is consistent with a recent increase in transposition rate. The *D. pulicaria* increase may have been triggered by insertion of *Pokey*A into a region of transcriptionally active rDNA. The expansion in *D. arenata* (thought to be of hybrid origin) may be a consequence of release from epigenetic repression following hybridization. Previous work found *D. obtusa* to be very different from the *D. pulex* complex; mean *Pokey*A is higher in rDNA (~75), rDNA array size is nearly twice as large (415), and the two are positively correlated. The predominance of *Pokey* in only one location could be explained by purifying selection against ectopic recombination between elements inside and outside rDNA.

**Electronic supplementary material:**

The online version of this article (doi:10.1186/s13100-016-0067-7) contains supplementary material, which is available to authorized users.

## Background

Transposable elements (TEs) are segments of DNA that can move around the genome. TEs are often detrimental to the host because they can interrupt gene function, their transposition has energy costs (e.g. the repair of double strand breaks), they can cause ectopic recombination [1], and the epigenetic mechanisms used to control them can shut down surrounding genes [2]. Usually, DNA transposons must undergo horizontal transfer to remain active [3]. However, *Pokey*, a class II cut-and-paste DNA transposon in the *piggyBac* superfamily [4] has been vertically inherited in the subgenus, *Daphnia* [5]. *Pokey* inserts at TTAA sites and creates a target site duplication (TSD). It ranges in size from 4.5 to 10 kilobase pairs (kb); the length variation is due to repeat sequences at the 5′ end derived from the ribosomal intergenic spacer (IGS) [4] and/or the ribosomal internal transcribed spacer (ITS) [6]. *Pokey* is the only DNA transposon known to insert in a specific location in ribosomal DNA (rDNA); other rDNA elements, such as R1 and R2, are non-Long Terminal Repeat (LTR) retrotransposons [7]. Nevertheless, Kojima and Jurka [8] recently reported the discovery of class II *Dada* TEs in organisms as taxonomically diverse as fish, molluscs, annelids, and cladoceran crustaceans (*Daphnia*). Like *Pokey*, they show target-site specificity for particular multicopy RNA genes (small nuclear RNA, transfer RNA), encode a putatively cut-and-paste transposase, and create TSDs on insertion. However, unlike other elements that target multicopy RNA genes, *Pokey* also inserts into TTAA sites outside rDNA in the genomes of North American (NA) *D. pulex* [9] and NA *D. pulicaria* [10].

In eukaryotes, rDNA is a multigene family arranged in tandem arrays of units each consisting of the 18S, 5.8S, and 28S rRNA genes plus the ITS, the external transcribed spacer (ETS), and the IGS. The number of rDNA units per eukaryotic genome varies from less than 50 to over 25,000 [11] and is usually much higher than the number required for viability [7]. rDNA typically displays the phenomenon of concerted evolution, which means that intraspecific sequence divergence between rDNA copies is generally much lower than interspecific divergence [7]. The mechanisms of concerted evolution are thought to be gene conversion and unequal crossing over [7], which can cause the number of rDNA units to fluctuate. Due to its multicopy nature and the presence of copies above the number required for viability, some TEs, such as R1, R2, and *Pokey*, have persisted in rDNA for very long periods of time [7] even though the homogenizing mechanisms of concerted evolution are expected to continually remove them. In addition, TEs inserted in rRNA genes render the genes non-functional, so selection is expected to favor loss of these elements [7].

*Daphnia* are freshwater cladoceran crustaceans with a worldwide distribution [12]. Based on molecular analyses, the *D. pulex* species complex (subgenus *Daphnia*) is composed of eight lineages between which hybridization frequently occurs: North American (NA) *D. pulex*, *D. melanica*, *D. middendorffiana*, North American (NA) *D. pulicaria*, *D. tenebrosa*, European (EU) *D. pulex*, European (EU) *D. pulicaria* [13], and *D. arenata* [14]. Although these analyses suggest that the North American and European populations of *D. pulex* and *D. pulicaria* should be classified as separate species, they have not been officially described as such [13]. While most cladocerans reproduce by cyclical parthenogenesis (diapausing eggs are produced sexually and direct-developing eggs are produced apomictically), populations of obligate parthenogens (diapausing eggs are also produced apomictically) also occur in some lineages of this species complex [13].

Eagle and Crease [10] found that the total haploid number of *Pokey* in NA *D. pulex* and NA *D. pulicaria* is usually less than 20 copies, most of which are found outside rDNA. In addition, *Pokey* number in rDNA is not correlated with rRNA gene number. In contrast, LeRiche et al. [15] found *Pokey* number in another species in the same subgenus, *Daphnia obtusa*, to be as high as 154 (mean = 75), almost all of which are located inside rDNA. Moreover, *Pokey* number in rDNA is strongly correlated with rRNA gene number.

Both autonomous (encoding a functional transposase) and non-autonomous (the transposase gene is degraded or partially deleted) copies of *Pokey* have been found [6]. Further, Miniature Inverted Repeat Transposable Elements (MITEs), which are very short (usually less than 600 nucleotides) [16], non-autonomous TEs have been observed in *D. arenata* [6]. Overall, Elliott et al. [6] discovered four TEs in the *D. arenata Pokey* family: the original *Pokey* group discovered in *D. pulex*, now called *Pokey*A; a variant containing a transposase gene (complete or partial) that was initially seen in *D. obtusa* [5], now called *Pokey*B; a MITE with terminal inverted repeats (TIRs) similar to those of *Pokey*A, called m*Pok*1, and a MITE with TIRs similar to those of *Pokey*B, called m*Pok*2. The m*Pok* elements average 760 base pairs (bp) in length [6]. *Pokey*A and m*Pok*1 have 16 bp imperfect TIRs, and *Pokey*B and m*Pok*2 have 12 bp imperfect TIRs [6]. Like *Pokey*A, *Pokey*B also contains an open reading frame (ORF) encoding a putative transposase and both ORFs contain an intron [6]. Mean sequence divergence between copies of *Pokey*A and *Pokey*B containing a complete or partial transposase gene from *D. arenata* is 40 %, suggesting that the two TE groups did not diverge from one another recently.

**Fig. 1:**
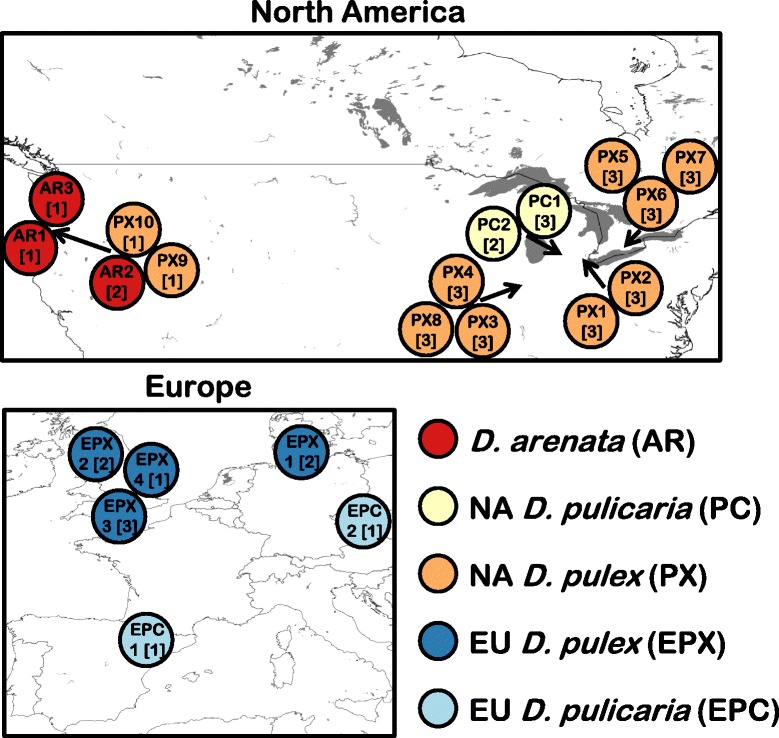
Location of *Daphnia* populations sampled. The numbers in square brackets indicate the number of isolates sampled from that population. NA = North America, EU = Europe

Three additional types of TEs have now been discovered in the *D. arenata Pokey* family, but we do not know if they occur in other lineages in the *D. pulex* complex. Our first goal was to determine the distribution of *Pokey* and m*Pok* in the *D. pulex* complex. We did this using quantitative polymerase chain reaction (qPCR) to estimate gene number in 45 cyclically parthenogenetic isolates representing five lineages: NA *D. pulex* (*N* = 26), NA *D. pulicaria* (*N* = 5), *D. arenata* (*N* = 4), EU *D. pulex* (*N* = 8), and EU *D. pulicaria* (*N* = 2) (Fig. 1, Additional file 1: Table S1). Our second goal was to test the hypothesis of recent transposition of *Pokey*A in NA *D. pulicaria*, suggested by Eagle and Crease [10], by estimating divergence among partial *Pokey*A sequences from four isolates; two with unusually high *Pokey*A number and two with numbers close to the average for this species.

## Results

### *Pokey* and m*Pok* number in the *D. pulex* complex

We used qPCR (Additional file 1: Table S2) to estimate the haploid number of the four TEs in the *Pokey* family (*Pokey*A, *Pokey*B, m*Pok*1, m*Pok*2) in the entire genome (t*Pokey*, tm*Pok*) and in rDNA (r*Pokey*, rm*Pok*) by comparing their PCR amplification rate to the PCR amplification rate of two single copy genes, *Gtp* (a member of the RAB subfamily of small GTPases) and *Tif* (a transcription initiation factor) [10], [17]. We calculated the number of *Pokey* and m*Pok* outside rDNA (g*Pokey*, gm*Pok*) by subtracting r*Pokey* or rm*Pok* from t*Pokey* or tm*Pok*. We also estimated the number of 18S and 28S rRNA genes in each isolate.

**Fig. 2:**
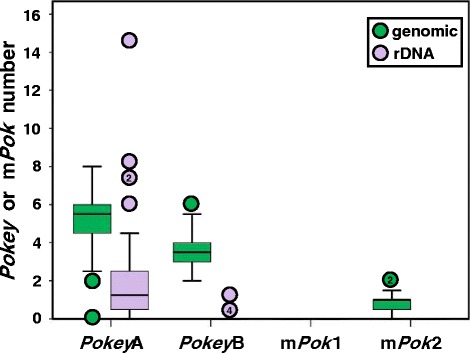
Box plot of haploid *Pokey* family number in 26 isolates of North American *Daphnia pulex. Pokey* or m*Pok* numbers greater or less than 1.5 times the box length are indicated with circles. The number within the circle indicates how many isolates have that particular number of *Pokey* or m*Pok*. Genomic = *Pokey* or m*Pok* inserted outside 28S, rDNA = *Pokey* or m*Pok* inserted in 28S

The mean haploid number of all *Pokey*A and * Pokey*B in NA *D. pulex* is 11.4 and never exceeds 19 copies per isolate (Additional file 1: Table S3). Mean g*Pokey*A plus g*Pokey*B number (8.6) exceeds mean r*Pokey*A plus r*Pokey*B number (2.8) and this is true for both *Pokey*A (5.3 vs 2.8) and *Pokey*B (3.6 vs 0.1, Fig. 2), individually. *Pokey*A number is higher than *Pokey*B number in NA *D. pulex*, inside and outside rDNA (Fig. 2). These patterns are similar in each of the 26 NA *D. pulex* isolates (Additional file 1: Table S3).

**Fig. 3:**
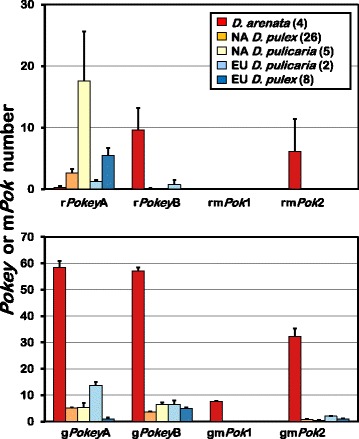
Haploid *Pokey* and m*Pok* number in 45 isolates from the *Daphnia pulex* complex. Black vertical bars are standard errors. NA = North America, EU = Europe, r*Pokey* = *Pokey* inserted in 28S, rm*Pok* = m*Pok*1 inserted in 28S, g*Pokey* = *Pokey* inserted outside 28S, gm*Pok* = m*Pok* inserted outside 28S

The mean total number of *Pokey*A plus *Pokey*B per haploid genome in three of the other four lineages (NA *D. pulicaria*, EU *D. pulex*, EU *D. pulicaria*) is similar to NA *D. pulex*, with means ranging from 11.4 to 29.4 (Fig. 3). In contrast, the mean total number in *D. arenata* is substantially higher (125.4) (Additional file 1: Table S3), and the mean number of g*Pokey*A plus g*Pokey*B is highest in *D. arenata* (Table 1, Fig. 3). Although the mean is lower compared to *D. arenata*, we still detected g*Pokey*B in all of the other 41 isolates, and g*Pokey*A in all but six of them (one NA *D. pulex*, one NA *D. pulicaria* and four EU *D. pulex*; Additional file 1: Table S3). The g*Pokey*A estimate in these six isolates was set to zero because the estimate of r*Pokey*A is larger than the estimate of t*Pokey*A giving a negative value for g*Pokey*A. Thus, we cannot exclude the possibility that very low numbers of g*Pokey*A occur in these isolates.

**Table 1:** Haploid number of *Pokey*, m*Pok*, and rRNA genes in isolates from the *Daphnia pulex* complex

		NA^c^ *D. pulex*	NA^c^ *D. pulicaria*	*D. arenata*	EU^c^ *D. pulex*	EU^c^ *D. pulicaria*
Gene^a^	N^b^	26	5	4	8	2
18S	Mean	216.3	310.4	303.8	194.5	366.3
	Std Dev^b^	85.3	112.7	38.3	93.2	35.7
	Range	112.5 to 440.5	176.0 to 460.0	248.0 to 332.5	97.5 to 339.5	341.0 to 391.5
28S	Mean	230.9	405.9	309.3	201.5	347.8
	Std Dev	81.1	128.3	30.3	124.3	9.5
	Range	119.5 to 452.5	234.5 to 576.5	264.5 to 330.5	87.5 to 418.0	341.0 to 354.5
r*Pokey*A	Mean	2.6	17.6	0.3	5.5	1.3
	Std Dev	3.4	17.9	0.5	3.4	0.4
	Range	0 to 14.5	3.5 to 48.0	0 to 1.0	1.0 to 10.5	1.0 to 1.5
r*Pokey*B	Mean	0.1	0	9.6	0	0.8
	Std Dev	0.3		7.0		1.1
	Range	0 to 1.0		5.0 to 20.0		0 to 1.5
rm*Pok*1	Mean	0	0	0	0	0
	Std Dev					
	Range					
rm*Pok*2	Mean	0	0	6.1	0	0
	Std Dev			10.6		
	Range			0 to 22.0		
g*Pokey*A	Mean	5.1	5.3	58.4	1.0	13.8
	Std Dev	1.7	4.0	4.9	1.3	1.8
	Range	0.0 to 8.0	0.0 to 10.5	52.5 to 63.5	0.0 to 3.0	12.5 to 15.0
g*Pokey*B	Mean	3.6	6.5	57.1	4.9	6.5
	Std Dev	1.1	1.5	2.8	1.2	2.1
	Range	2.0 to 6.0	5.0 to 9.0	54.5 to 61.0	3.5 to 7.0	5.0 to 8.0
gm*Pok*1	Mean	0	0	7.5	0	0
	Std Dev			0.7		
	Range			7.0 to 8.5		
gm*Pok*2	Mean	0.8	0.4	32.3	1.0	2.0
	Std Dev	0.5	0.4	6.1	0.4	0.0
	Range	0 to 2.0	0 to 1.0	25.0 to 38.5	0.5 to 1.5	2.0 to 2.0

^a^18S = 18S rRNA genes, 28S = 28S rRNA genes, r*Pokey* = *Pokey* inserted in 28S, g*Pokey* = *Pokey* inserted outside 28S, rm*Pok* = m*Pok* inserted in 28S, gm*Pok* = m*Pok* inserted outside 28S

^b^N = number of isolates. Std Dev = Standard Deviation

^c^NA = North American, EU = European

The mean number of r*Pokey*A is lowest in *D. arenata* (Table 1, Fig. 3) and highest in NA *D. pulicaria*. The high mean in NA *D. pulicaria* is due to the unusually high number in two isolates (PC1.2, PC2.1), as previously described by Eagle and Crease [10]. These isolates were chosen to determine whether the high number of r*PokeyA* could be explained by recent transposition (see below). r*Pokey*A is absent in three of the four *D. arenata* isolates, but is only absent in three of the other 41 isolates (Additional file 1: Table S3). This pattern is reversed for r*Pokey*B in which the mean number is highest in *D. arenata* (Table 1, Fig. 3), but the element is absent from rDNA in 35 of the other 41 isolates (Additional file 1: Table S3).

The MITEs, m*Pok*1 and m*Pok*2 are primarily found in *D. arenata* (Fig. 3). Indeed, the only MITE we detected in the other four lineages is gm*Pok*2, but it is present in no more than 2 copies per haploid genome (Table 1), and absent in most isolates (Additional file 1: Table S3). m*Pok*2 was detected both inside (mean = 6) and outside (mean = 32) rDNA in *D. arenata* (Table 1, Fig. 3), but m*Pok*1 was only detected outside rDNA (mean = 7.5) (Table 1, Fig. 3).

### rRNA gene number in the *D. pulex* complex

**Fig. 4:**
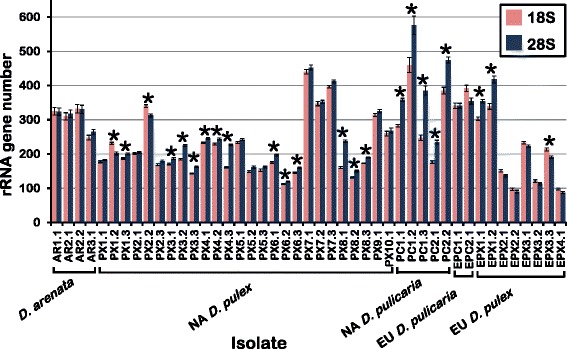
Haploid 18S and 28S rRNA gene number in isolates from the *Daphnia pulex* complex. * means are significantly different at the 5 % level after sequential Bonferroni correction. 18S = 18S rRNA genes, 28S = 28S rRNA genes. Each isolate is identified by a 2 or 3-letter lineage code followed by x.y, where x is the population and y is the isolate. NA = North America, EU = Europe

Haploid 18S number ranges from 98 to 460 and 28S number ranges from 88 to 577 in the 45 *Daphnia* isolates (Table 1). 28S number is larger than 18S number by 2.5 to 138 copies in 33 of the 45 isolates, and the difference is significant in 23 of these (Fig. 4, Additional file 1: Table S3). Despite the differences between the two genes, their numbers are significantly correlated in NA *D. pulex* with a slope of 0.92 and an R^2^ of 0.937 (*p* < 10^−15^, Table 2, Additional file 2: Figure S1). The slope of the correlation based on the other 19 isolates is 1.19 with an R^2^ of 0.873 (*p* = <10^−9^, Table 2, Additional file 2: Figure S1). There is no correlation between 28S and total r*Pokey* plus rm*Pok* number in the 26 NA *D. pulex* isolates (R^2^ = 0.04, Table 2, Additional file 2: Figure S2). Similarly, there is no correlation between 28S and r*Pokey* plus rm*Pok* number in the 19 isolates from the other four lineages (R^2^ = 0.03, Table 2, Additional file 2: Figure S2).

**Table 2:** Correlation between haploid r*Pokey* family and rRNA gene number in isolates from five lineages of the *Daphnia pulex* complex

lineage	X-axis^c^	Y-axis^c^	slope	y-intercept	R^2^	*p*-value*	Figure
NA *D. pulex* ^a^	18S	28S	0.920	31.88	0.937	**6.3 x 10** ^**−16**^	Additional file 2: Figure S1
four lineages^b^	18S	28S	1.19	23.39	0.873	**4.9 x 10** ^**−9**^	Additional file 2: Figure S1
NA *D. pulex*	28S	r*Pokey* family	−0.008	4.60	0.035	0.361	Additional file 2: Figure S2
four lineages	28S	r*Pokey* family	0.014	6.44	0.026	0.51	Additional file 2: Figure S2

^a^This analysis is based on 26 isolates

^b^This analysis is based on 19 isolates including *D. arenata* (4), EU *D. pulex* (8), and NA (5) and EU (2) *D. pulicaria*

^c^18S = 18S rRNA genes, 28S = 28S rRNA genes, r*Pokey* family = *Pokey* plus m*Pok* inserted in 28S = [r*Pokey*A + r*Pokey*B + rm*Pok*1 + rm*Pok*2]

*p-values < 0.05 are indicated in bold font

### Variation in partial *Pokey*A sequences in North American *D. pulicaria*

An approximately 1500 bp segment, including 519 bp of the 5′ end of the *Pokey*A transposase and the length-variable region upstream was PCR-amplified, cloned, and sequenced from four isolates of NA *D. pulicaria*. We predicted that sequence divergence would be lower among copies from isolates with high *Pokey*A number if the increase was a consequence of recent transposition. Using data from Eagle and Crease [10], we chose two isolates, one with high and one with average r*Pokey*A number, from each of two populations. The number of other *Pokey* family elements is similar in all four isolates (Table 3). The number of 28S genes in these isolates ranges from 235 to 577 and the one with the lowest number (PC2.1) has the highest number of r*Pokey*A (48, Table 3).

**Table 3:** Haploid number of *Pokey*, m*Pok*, and rRNA genes in four isolates of North American *Daphnia pulicaria*

	PC1.1	PC1.2	PC2.1	PC2.2
gene^a^	Average 1^b^	High 1	High 2	Average 2
18S	283	460	176	385.5
28S	358.5	576.5	234.5	474.5
r*Pokey*A	6	17	48	3.5
g*Pokey*A	3.5	7.5	0	5
g*Pokey*B	5	6.5	9	6
gm*Pok*2	1	0	0.5	0.5
mean number of differences from consensus^c^	49.9	10.8	1.6	45.8
standard deviation	43.3	36.9	3.4	32.5
range	0 to 97	0 to 128^d^	0 to 6	12 to 78
*p*-value^e^		0.027		0.010

^a^18S = 18S rRNA genes. 28S = 28S rRNA genes. r*Pokey*B, rm*Pok*2 and m*Pok*1 were not detected in these four isolates

^b^Average and High correspond to the sequence labels in Fig. 4

^c^The number of differences between the consensus and each of 12 cloned 1500 bp sequences was determined for each isolate. Gaps were included in the analysis. Consecutive gaps were counted as one variant nucleotide position

^d^All other values were 0 or 1 for this isolate

^e^ANOVA was used to compare the mean number of differences across all four isolates. This test was significant (F = 6.62, d.f. = 3, *p* = 0.0009). A *Post hoc* Tukey's HSD test was used to determine if means in the Average and High isolates from the same population were significantly different. They p-values refer to the Tukey’s tests

**Fig. 5:**
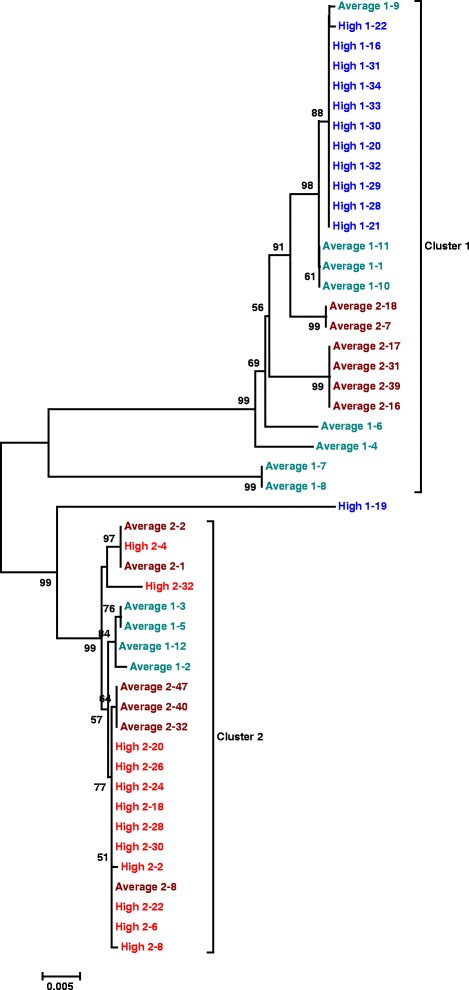
Dendrogram of partial *Pokey*A sequences from four isolates of North American *Daphnia pulicaria*. The isolates were from two different populations, PC1 and PC2. Two isolates possessed High numbers of *Pokey*A, PC1.2 (blue) and PC2.1 (red); and two isolates possessed Average numbers of *Pokey*A, PC1.1 (teal) and PC2.2 (dark red). The sequences were approximately 1500 bp in length and included 519 bp of the 5′ end of the *Pokey*A transposase and the length-variable region upstream. Bootstrap values >50 from 1000 replicates are provided on the nodes

On average, the mean number of differences between each cloned sequence and the isolate consensus sequence is lower in the isolate with a high *Pokey*A number compared to the isolate with an average number from the same population. Moreover, the difference between means is significant in both populations (Table 3). The Neighbor-joining dendrogram of these sequences (Fig. 5) shows two major clusters; cluster 1 contains 11 of 12 sequences from isolate High 1 (PC1.2), as well as sequences from both Average isolates. Cluster 2 contains all 12 sequences from isolate High 2 (PC2.1), as well as sequences from both Average isolates. All but two of the sequences encode a putatively functional transposase (no insertions, deletions, or stop codons). The exceptions are High 1–19, which contains several stop codons and numerous nucleotide substitutions throughout, and Average 2-32, in which the first 29 bp of the coding sequence are deleted (gaps are not considered in calculations of sequence divergence so this sequence does not appear to be divergent from other sequences in cluster 2).

## Discussion

### *Pokey* family distribution in NA and EU *D. pulex* and *D. pulicaria*

In general, *Pokey* number is similar in four of the five lineages we examined; NA and EU *D. pulex* and NA and EU *D. pulicaria*. The mean number of *Pokey*A plus *Pokey*B is less than 20 and both TEs are primarily located outside of rDNA. Our *Pokey*A numbers (Table 1) are similar to estimates from Eagle and Crease [10] for NA *D. pulex* (mean r*Pokey*A = 2.1 and mean g*Pokey*A = 9.6 based on 43 isolates) and NA *D. pulicaria* (mean r*Pokey*A = 6.6 and mean g*Pokey*A = 9.5 based on 26 isolates). However, the NA *D. pulicaria* r*Pokey*A mean in the current study (17.6) is higher than the previous estimate because we only included five isolates, two of which have unusually high r*Pokey*A number, and were chosen for that reason.

Both the current and previous estimates of g*Pokey*A and g*Pokey*B are within the range of other DNA transposons found in the *Daphnia* genome sequence [18]. For example, there are approximately 31 copies per *Tc1/mariner* family (217 copies from 7 families) and approximately 6 copies per *hAT* family (33 copies from 6 families). In contrast, the mean numbers of r*Pokey*A and r*Pokey*B (Table 1) tend to be low compared to the mean number of rDNA-specific non-LTR retroelements, R1 (34) and R2 (12) in *D. melanogaster* [19].

Overall, *Pokey*A outnumbers *Pokey*B, which could be due to a higher transposition rate, weaker purifying selection at the level of the host, and/or lower rates of deletion of *Pokey*A. Due to the fact that they both insert into the same TTAA site in rDNA, it seems unlikely that the strength of purifying selection against these two TEs at the level of the host, or their rate of deletion differs. Therefore, we suggest that the higher number of *Pokey*A may be due to a higher transposition rate, which could be tested using yeast excision assays.

Although *Pokey* number and distribution is similar in the four *D. pulex* and *D. pulicaria* lineages, it was necessary to set g*Pokey*A to 0 in six isolates because r*Pokey*A exceeds t*Pokey*A. The t*Pokey*A primer pair is located at the 5′ end of the transposase gene, and can only amplify autonomous *Pokey*A. Conversely, it was necessary to locate the r*Pokey*A forward primer near the 3' end of the gene, even though it was not possible to design primers in that region to target only r*Pokey*A or rm*Pok*1. Consequently, the r*Pokey*A forward primer amplifies both autonomous and non-autonomous r*Pokey*A, as well as rm*Pok*1. However, the higher number of r*Pokey*A compared to t*Pokey*A cannot be due to the presence of rm*Pok*1 because no copies of m*Pok*1 were detected in these isolates by end-point PCR using internal, m*Pok*1-specific primers. Thus, the higher number of r*Pokey*A compared to t*Pokey*A in some isolates is most likely explained by copies of r*Pokey*A that did not amplify with the t*Pokey*A primers (Table 1, Fig. 3), but further study would be required to confirm this.

The number of m*Pok* we observed in NA and EU *D. pulex* and NA and EU *D. pulicaria* is very low (0 to 2 haploid copies per isolate) compared to other *piggyBac* MITEs, for which numbers range from 50 to 2x10^4^ in metazoans including nematodes, insects, and vertebrates [20]. Indeed, the absence of any m*Pok*1 in the 41 isolates we examined suggests that this MITE may not occur in these four lineages. In addition, the absence of m*Pok*2 in rDNA and the low number outside rDNA (maximum of two copies) suggests that the activity of m*Pok*2 is very low in these lineages.

### *Pokey* family distribution in *D. arenata*

While m*Pok*1 and m*Pok*2 were both detected in *D. arenata*, the former was only detected outside rDNA and in low numbers suggesting its activity is low. The mean p-distance between copies of m*Pok*1 and the m*Pok*1 consensus sequence is only 2 % in *D. arenata* isolate AR1.1 (Additional file 2: Figure S3). This, along with the fact that it does not occur in the other lineages, suggests that m*Pok*1 could be of recent origin. In contrast, m*Pok*2 occurred both inside and outside rDNA, with a mean haploid number of genomic copies greater than 30. This is at the top of the range for g*Pokey* in the other four lineages (Fig. 3), suggesting that m*Pok*2 may be active in *D. arenata.* Recent activity is also consistent with a p-distance less than 3 % between the m*Pok*2 consensus sequence and 75 % of the m*Pok*2 in isolate AR1.1 (Additional file 2: Figure S3). However, the p-distance ranges from 4 to 22 % for the other copies of m*Pok*2 (Additional file 2: Figure S3). This, and the fact that m*Pok*2 is found in other lineages in the *D. pulex* complex, suggests that this MITE is not of recent origin [6]. Further work is required to determine the distribution of m*Pok* in the *D. pulex* complex, and in other species in the subgenus *Daphnia*.

The number of *Pokey* and m*Pok*, except r*Pokey*A, is substantially higher in *D. arenata* than the other four lineages, resulting in a very different distribution (Fig. 3). *Daphnia arenata* is endemic to western Oregon [13] and may have diverged from NA *D. pulex* during the Pleistocene glaciation, during which it was isolated from other refugial populations south of the glaciers by the Cordilleran ice sheet [21]. This restriction to a small geographic region could have resulted in a smaller effective population size, such that the fate of slightly deleterious mutations (such as some TE insertions) could have been primarily determined by genetic drift instead of purifying selection [22].

Another explanation for the high *Pokey* number in *D. arenata* is a much higher transposition rate compared to the other lineages. Such an increase could be the result of changes in *Pokey,* such as mutations in the transposase that increase transposition rate, or mutations in the terminal repeats that increase recognition by the transposase. These hypotheses could be tested using yeast excision, yeast one-hybrid, and/or electrophoretic mobility shift assays.

A third explanation for high *Pokey* number in *D. arenata* is a loss of epigenetic regulation by the host. The most well-known example of such a loss is the phenomenon of P-M hybrid dysgenesis in *Drosophila*, which occurs when a male from a strain with *P*-elements is mated to a female from a strain without *P*-elements [23]. The female usually provides the epigenetic regulation, but if she does not have *P*-elements, then the paternal *P*-elements are able to transpose without regulation in the offspring [24], leading to effects such as male recombination, sterility, mutation, chromosomal aberrations, and nondisjunction [23].

Interspecific hybridization is also known to stimulate increased TE activity due to the disruption of host defenses, although this is not always the case (see Ungerer and Kawakami [25] for an example). Labrador et al. [26] found that the *Osvaldo* LTR retrotransposon has a higher transposition rate in hybrids of *Drosophila buzzatii* and *Drosophila koepferae* compared to non-hybrids. Similar increases in transposition rate in interspecific hybrids have been seen for other elements in rice and *Drosophila* (e.g. [27–31]).

Vergilino et al. [32] suggested that *D. arenata* may be a hybrid between NA *D. pulex* and *D. tenebrosa* or EU *D. pulicaria* because the position of *D. arenata* varies on phylogenetic trees generated from different nuclear loci [32–34]. NA *D. pulex* is likely the maternal parent based on analysis of mitochondrial DNA [13, 21]. If *D. arenata* does have a hybrid origin, release from host regulation could explain the relatively high number of *Pokey* and m*Pok*2 and possibly the occurrence of m*Pok*1. Further work is required to establish the distribution of m*Pok* in other *Daphnia* species, determine the origin of m*Pok*1, confirm the hybrid origin of *D. arenata*, and test the hypothesis that the high *Pokey* load in *D. arenata* is a consequence of increased transposition in a hybrid lineage. If this is the case, we would also expect the number of active TEs from other superfamilies to be higher in *D. arenata* compared to other lineages in the *D. pulex* complex, but this remains to be tested.

### Distribution of *Pokey* in the subgenus, *Daphnia*

Our results are very different from those obtained for *D. obtusa* in which r*Pokey* number is much higher (mean = 75 for *Pokey*A and 9 for *Pokey*B) and g*Pokey* number is much lower (mean = 0.5 for *Pokey*A) [15]. Moreover, r*Pokey*A is significantly positively correlated with 28S number in *D. obtusa* (R^2^ = 0.54). The mean haploid rRNA gene number in lineages of the *D. pulex* complex is 240, with values ranging from 90 to nearly 580. Conversely, the mean haploid number in 21 isolates of *D. obtusa* is 415, with values ranging from 180 to 960. LeRiche et al. [15] suggested that r*Pokey*A number could be higher in *D. obtusa* due to the larger size of its rDNA array, which may be a consequence of higher rates of sister chromatid exchange [15]. A large rDNA array provides more *Pokey* insertion sites, and a high rate of sister chromatid exchange provides more opportunity for the element to increase via recombination, even if the transposition rate is low.

Although the number of *Daphnia* species whose *Pokey* distribution has been studied is modest, the emerging pattern is that these elements tend to be concentrated in one genomic location (inside or outside of rDNA) and occur in low number in the other. In the *D. pulex* complex, *Pokey* is concentrated outside rDNA and in *D. obtusa* it is concentrated inside rDNA. Moreover, with the exception of *D. arenata*, g*Pokey* in the *D. pulex* complex (less than 30 copies) tends to be much lower than r*Pokey* in *D. obtusa* (up to 154 copies). Even so, the lower number of g*Pokey* in the *D. pulex* complex is consistent with the number of other DNA elements observed in the *Daphnia* genome sequence as mentioned above [18].

One explanation for the tendency of *Pokey* to be concentrated in only one genomic location is purifying selection against chromosomal rearrangements caused by ectopic recombination between copies inside and outside rDNA. The ectopic recombination model [35] suggests that TEs should accumulate in genomic regions of low recombination due to the deleterious effects of exchange between elements at non-homologous sites. Although we do not know the location of g*Pokey* in *Daphnia* genomes, their relatively low number suggests that their accumulation is resisted by selection. In contrast, Eickbush [36] suggested that ectopic recombination between TEs inserted in rDNA may not have severe effects because the outcome is similar to the effects of concerted evolution. On the other hand, ectopic recombination between g*Pokey* and r*Pokey* could severely impact genome organization and result in strong purifying selection, such that *Pokey* is generally not able to persist in substantial numbers in both locations in the same genome. *Daphnia arenata* is a notable exception to this general pattern as *Pokey*B and m*Pok*2 numbers are unusually high both inside and outside rDNA. If *Pokey* has been recently transposing in *D. arenata,* as suggested above, then there may not have been sufficient time for purifying selection to limit the expansion of *Pokey* in one or the other genomic location.

### Variation among partial *Pokey*A sequences in North American *D. pulicaria*

Eagle and Crease [10] suggested that the transposition rate of r*Pokey*A may have recently increased in some populations of NA *D. pulicaria* because its number is unusually high in some isolates from populations PC1 and PC2. Sequence divergence between TE copies that have recently transposed is expected to be low as there has been little time for them to accumulate differences from their parent copy. Our results are consistent with this hypothesis as the sequences from isolates with high *Pokey*A number (PC1.2 and PC2.1) show significantly less deviation from their consensus sequence than do sequences from isolates with average *Pokey*A numbers in the same population (PC1.1 and PC2.2, Table 3). Moreover, all but two of the sequences encode a potentially functional transposase. However, we cannot rule out the possibility that recent recombination within the rDNA array has also played a role in this r*Pokey*A expansion.

A recent increase in transposition rate is often a consequence of an element’s ability to evade host silencing, which has been suggested to depend on the distribution of elements that occur in rDNA. For example, Eickbush et al. [37] found that rates of R2 transcription are higher when the elements are spread throughout the rDNA array than when they are clustered, and indirect evidence suggests that *Pokey* insertions are clustered in the rDNA of NA *D. pulex* [38]. Eickbush et al. [37] concluded that the largest continuous block of uninserted rDNA units tend to be transcribed, while other units remain silent. However, when R2 insertions are spread across the rDNA array, or have recently transposed into the midst of a large continuous block of uninserted units, they are more likely to be transcribed. Similarly, it is possible that recent insertion of *Pokey*A into a large continuous block of uninserted rDNA units in a NA *D. pulicaria* individual (either by recombination or transposition) triggered an increase in *Pokey*A transcription. The hypothesis that transcription rate is higher in individuals with high *Pokey*A number could be tested using nuclear run-on transcription assays and Reverse Transcription-PCR [37]. In addition, fiber-FISH assays could be used to determine the arrangement of *Pokey* within rDNA arrays. This technique has been used to show that *Pokey* is dispersed in isolate AR1.1 (Figure S11 in [18]).

## Conclusions

The goals of this study were to determine the distribution of the *Pokey* family (*Pokey*A, *Pokey*B, m*Pok*1 and m*Pok*2) inside and outside rDNA in lineages of the *D. pulex* complex, and to test the hypothesis that r*Pokey*A expansion in NA *D. pulicaria* is the result of recent transposition. We found the distribution to be similar in four of the lineages; *D. pulex* and *D. pulicaria* from North America (NA) and Europe (EU) and in general, *Pokey* is more common outside than inside rDNA. *Pokey*A expansion in NA *D. pulicaria* rDNA appears to be recent and we suggest it could have been triggered by a change in rDNA distribution that reduced the host's ability to regulate *Pokey* transcription.

The *Pokey* family distribution in *D. arenata* is very different from the other four lineages. In particular, the mean number of both *Pokey*A and *Pokey*B outside rDNA is five to six times higher in *D. arenata* and the two types of m*Pok* are nearly exclusive to this lineage. We suggest that the proliferation of *Pokey* and m*Pok* in *D. arenata* may be a consequence of release from epigenetic repression as a result of interspecific hybridization, although a hybrid origin for this lineage requires confirmation.

The mean number of rRNA genes and r*Pokey*A is much larger in *D. obtusa* compared to the five *D. pulex* lineages. However, both *Pokey*A and *Pokey*B are absent or nearly so outside *D. obtusa* rDNA. Overall, these results suggest that *Pokey* primarily occupies only one or the other genomic location within a species. We suggest that this may be a consequence of purifying selection against ectopic recombination between elements in different genomic locations, which could cause severe genomic rearrangements.

Elliott et al. [6] suggested that *Pokey* originated as a genomic element and subsequently invaded *Daphnia* rDNA, which makes it unique compared to other DNA elements. Moreover, *Pokey* has been vertically inherited in the subgenus *Daphnia* since it diverged from the other *Daphnia* subgenera, *Ctenodaphnia* and *Hyalodaphnia* [10], which may have occurred as long ago as 100 million years [39]. It seems likely that the persistence of *Pokey* in *Daphnia* over such a long period of time is at least partially due to the potential for copies from one genomic location to reinvade the other if it is lost [6]. However, the factors that determine whether *Pokey* is primarily a genomic or an rDNA element within each species, and whether this configuration is stable over time are unknown and warrant further research.

## Methods

### *Daphnia* isolates and DNA extractions

We analyzed a total of 45 cyclically parthenogenetic *Daphnia* isolates from five lineages in the *D. pulex* complex (Fig. 1, Additional file 1: Table S1). Twenty-six of these isolates were NA *D. pulex.* One to three isolates were sampled from ten populations in the Midwest United States and southern Ontario. In addition, 19 isolates from the other lineages were included: four *D. arenata*, five NA *D. pulicaria*, eight EU *D. pulex*, and two EU *D. pulicaria. Daphnia* individuals were collected and clonally propagated (isolates) as described in Eagle and Crease [10]. DNA was extracted from multiple individuals from each isolate using phenol:chloroform or the Aquagenomics kit (MultiTarget Pharmaceuticals LLC, Salt Lake City, Utah, USA) as in Eagle and Crease [10]. DNA concentrations, which ranged from 13 to 4400 ng/μL (Additional file 1: Table S1), were measured using a NanoDrop® ND-8000 spectrophotometer (ThermoScientific).

### Estimation of *Pokey* family and rRNA gene number

We used the SYBR green real-time qPCR and ΔC_T_ relative quantification method as described in Eagle and Crease [10] to estimate the haploid number of each type of gene. C_T_, or cycle threshold, is the point at which the amplification curve crosses a set threshold. We estimated the number of the four TEs in the *Pokey* family (*Pokey*A, *Pokey*B, m*Pok*1, and m*Pok*2) in the entire genome (t*Pokey*, tm*Pok*) and in rDNA (r*Pokey*, rm*Pok*), as well as the number of 18S and 28S rRNA genes in all 45 *Daphnia* isolates.

A total of 11 primer pairs were used (Table 4). The forward primers for r*Pokey*/rm*Pok* are located near the 3’ end of the element and the reverse primer is located in the 28S rRNA gene downstream of the *Pokey* family insertion site. Standard curves were run to determine the percent amplification efficiency (PAE) for each primer pair (Table 4, Additional file 3).

**Table 4:** Primer pairs used for qPCR of the *Pokey* family and rRNA genes in the *Daphnia pulex* complex

Gene^a^	Primer	Primer sequence	Amplicon Size (bp)	Source^b^	Threshold^c^	PAE^d^
18S	18S 1864 F	5'-ccg cgt gac agt gag caa ta	50	[17]	0.200	0.947
	18S 1913 R	5'-ccc agg aca tct aag ggc atc		[17]		
28S	28S 2508 F	5'-gcc tgc tcg tac cga tat cc	50		0.200	0.937
	28S 2558 R	5'-cta gag gct gtt cac ctt gga ga				
t*Pokey*A	Pok 3720 F	5'-cag ttc aaa gag tgg ctc ctc c	50		0.200	0.868
	Pok 3770 R	5'-cgg gtc tga ctt ctg gtt cg				
t*Pokey*B	PokB 1375 F	5'-aaa gag gag aag aat gac ccg g	50		0.200	0.897
	PokB 1425 R	5'-tca gaa gag cac cct acc ttg g				
tm*Pok*1	mPok1 524 F	5'-gga cac cta tgg cgg gat t	50		0.200	0.874
	mPok1 574 R	5'-cgc tga ggt ctg tcg gga				
tm*Pok*2	mPok2 714 F	5'-ggt cag ttg gct ccg aca a	45		0.185	0.911
	mPok2 759 R	5'-aa ccc ttt atc gac gcg aag a		T. Elliott, unpublished		
r*Pokey*A + rm*Pok*1	Pok 6561 F	5'-caa tcg aat ccg acc atc g	66		0.237	0.914
	28S 3073 R	5'-tga cga ggc att tgg cta cc				
r*Pokey*B	PokB 4283 F	5'-aat ttc agt caa gca cgg cc	70		0.244	0.903
	28S 3073 R	5'-tga cga ggc att tgg cta cc				
rm*Pok*2	mPok 714 F	5'-ggt cag ttg gct ccg aca a	68		0.240	0.900
	28S 3073 R	5'-tga cga ggc att tgg cta cc				
*Tif*	TIF392F	5'-gac atc atc ctg gtt ggc ct	50	[17]	0.200	0.936
	TIF442R	5'-aac gtc agc ctt ggc atc tt		[17]		
*Gtp*	GTP385R	5'-tat tca gca tgg aga gac ggc	50	[17]	0.200	0.928
	GTP435R	5'-gat gtc gac tga cgc tgg aa		[17]		

^a^18S = 18S rRNA genes, 28S = total 28S rRNA genes, t*Pokey* = total *Pokey*, tm*Pok* = total m*Pok*, r*Pokey* = *Pokey* inserted in 28S, rm*Pok* = m*Pok*1 inserted in 28S

^b^Unless otherwise indicated, primers were designed for this study

^c^A threshold of 0.2 was used for 50 bp amplicons. The threshold for larger amplicons was determined using the formula, 0.2 x 2^[1-(50/length in bp)] as in Eagle and Crease [10]

^d^PAE = primer amplification efficiency

qPCRs had a final volume of 20 μL and contained 1X PerfeCTa SYBR Green FastMix, ROX (Quanta BioSciences) and 0.25 μM of each primer. Reactions were run on a StepOne Plus instrument (Applied Biosystems) using the following protocol: 95 °C for 10 min; followed by 40 cycles of 95 °C for 5 s and 60 °C for 30 s. Melt curves were run by heating the amplicons at 95 °C for 15 s, decreasing the temperature to 60 °C for 1 min, then heating to 95 °C in 0.3 °C increments. The DNA template was either serial dilutions of amplicons generated from plasmid DNA (standard curve plates) or 10 ng of genomic DNA (experimental plates). Samples were run in triplicate with the exception of the tm*Pok*1 and rm*Pok*2 primer pairs for samples in which previous end-point PCR produced no amplicon (Additional file 3). In such cases, only a single reaction was done. Negative control reactions were run for each primer pair on every standard curve plate. Negative control reactions were also run for each primer pair on 15 out of the 22 experimental plates (Additional file 3). The StepOne Software (Applied Biosystems) set the baseline for each reaction. The threshold was set based on amplicon size as in Eagle and Crease [10] (Table 4). A threshold of 0.2 was used for 50 bp amplicons, and the threshold for larger amplicons was determined using the formula, 0.2 x 2^[1-(50/length in bp)].

C_T_ values were used to estimate gene number, using the formula: 2^-ΔCT^ where ΔC_T_ is ([C_T_ x PAE _multicopy gene_] - [C_T_ x PAE _single-copy gene_]), as in Eagle and Crease [10]. If the standard deviation for mean C_T_ for a gene was greater than 0.2, then the most extreme value was omitted (Additional file 1: Table S2) from further analysis. If there was no clear outlier among the three C_T_ values, then all three values were used. The two or three C_T_ values for each multicopy gene were compared to the two or three C_T_ values for both single copy genes producing 8 to 18 estimates of gene number. The *Tif*-*Gtp* ratio was 1.66 in isolate PX1.2 and 1.42 in isolate PX2.2; these two isolates likely have three copies of the *Tif* gene instead of the expected two. Therefore, the estimates of gene number using *Tif* as the single copy gene were multiplied by 1.5 in these isolates. In addition, both tm*Pok*1 and r*Pokey*A + rm*Pok*1 amplified in one isolate, AR3.1. To determine if the estimate of r*Pokey*A + rm*Pok*1 included r*Pokey*A, rm*Pok*1 or both, this isolate was screened for rm*Pok*1 with end-point PCR (Additional file 3), but no amplicon was detected.

g*Pokey*/gm*Pok* number was calculated as [t*Pokey* – r*Pokey* or tm*Pok* – rm*Pok*]. In six isolates (EPX1.1, EPX1.2, EPX2.1, EPX2.2, PC2.1, and PX6.3), the calculation of g*Pokey*A produced a negative number in which case g*Pokey*A was set to zero, and t*Pokey*A was assumed to be the same as r*Pokey*A.

We used paired t-tests for means, two-sample t-tests assuming unequal variances with the sequential Bonferroni correction [40], and linear regressions (Microsoft Excel, Richmond, Washington, USA) to examine the relationship between 18S and 28S rRNA gene number. We also estimated the correlation between rRNA genes and *Pokey* family number using regression analysis.

### Sequencing partial *Pokey*A copies from North American *D. pulicaria*

We cloned and sequenced partial *Pokey*A elements from four NA *D. pulicaria* isolates from two populations. From each population, an isolate with high number of *Pokey*A (PC1.2, PC2.1) and an isolate with average number of *Pokey*A (PC1.1, PC2.2) were selected (Table 3). The average number of *Pokey*A for NA *D. pulicaria* was based on the results of Eagle and Crease [10]. Partial *Pokey*A sequences were amplified from approximately 50 ng of genomic DNA in a 25 μL reaction, which contained 1X Phusion HF Buffer (NEB), 0.4 mM dNTPs, 0.08 μM each of the Pok-2904 F (5’ ggg aca tag gtg tcc cgg) and Pok-6178R (5’ tcg acc agg ggt ctt tcc agt c) primers, and 1 units of Phusion DNA Polymerase. Reactions were run on either a PTC-100 Thermocycler (MJ Research) or a T100 Thermocycler (Bio-Rad Laboratories, Inc.) using the following protocol: 3 min initial denaturation at 98 °C; 35 cycles of 10 s denaturation at 98 °C, 30 s annealing at 55 °C, and 2 min elongation at 72 °C; followed by a final elongation at 72 °C for 10 min. The 3.3 kb amplicons were verified by electrophoresis on a 1 % TAE agarose gel, stained with GelRed™ Nucleic Acid Gel Stain (Biotium, Inc.) and visualized under UV light. The amplicons were then cloned into the plasmid, pSC-B-amp/kan using the StrataClone Blunt PCR Cloning Kit (Agilent Technologies) as per the manufacturer’s protocol with a few modifications. The modifications were as follows: 2.5 μL of PCR product and 0.5 μL of StrataClone Blunt Vector Mix were used in the ligation; 25-50 μL of StrataClone SoloPack cells was used for the transformation; an incubation of 30–45 min was used because the insert was large (3.3 kb); 500 μL of Terrific Broth was used instead of 250 μL of LB medium; and 50 μL and 400 μL were plated instead of 5 μL and 100 μL.

Positive colonies (white) were triple-streaked onto new plates and grown overnight. One of the three streaks was added with a toothpick to 10 μL of water and incubated at 99.9 °C for 3 min. One microliter was amplified in a 25 μL reaction with two sets of primers: Pok-2904 F with Pok-3811R (5’ ccg tgt tac ttc acc atc gg) to generate a 910 bp fragment; and Pok-3720 F (5’ cag ttc aaa gag tgg ctc c) with Pok-4488R (5’ gaa tcg ctc gcg agt cat gg) to generate a 770 bp fragment. Reactions contained 1X GenScript buffer (GenScript USA Inc.), 0.04 mM dNTPs, 0.04 μM of each primer, and 0.5 units of GenScript DNA polymerase (GenScript USA Inc.). Reactions were run on either a PTC-100 Thermocycler (MJ Research) or a T100 Thermocycler (Bio-Rad Laboratories, Inc.) using the following protocol: 2 min initial denaturation at 94 °C; 35 cycles of 30 s denaturation at 94 °C, 30 s annealing at 55 °C, and 1 min extension at 72 °C; followed by a final elongation at 72 °C for 5 min. Amplicons from 12 clones per isolate were sequenced with the primers Pok-2904 F and Pok-3720 F in 12 μL reactions. The reactions contained 0.3 μL BigDye® Terminator v3.1 Ready Reaction Mix (Applied Biosystems), 0.4X Sequencing Buffer (Applied Biosystems), 0.83 μM of primer, and 2 μL of amplicon. Sequencing reactions were run for 1 min at 96 °C followed by 30 cycles of a 20 s denaturation at 96 °C, a 20 s annealing at 55 °C, and a 4 min extension at 60 °C. Reactions were resolved on an ABI 3730 DNA Analyzer (Applied Biosystems) by the Genomics Facility at the University of Guelph. Sequences were analyzed using CLC Main Workbench software (CLC Bio). We used MEGA 5.0 [41] to generate a 1608 bp alignment containing 48 sequences from the four NA *D. pulicaria* isolates. The maximum composite likelihood model was used to estimate a matrix of pairwise sequence divergence from which a Neighbor-Joining dendrogram was generated. Gaps were excluded from this analysis using the pairwise deletion option. In addition, a consensus sequence was generated for each isolate from the 12 sequences. The number of differences between each sequence and its consensus was determined in MEGA. Gaps were included in this analysis, but consecutive gaps were coded as single nucleotide changes. ANOVA was used to test for heterogeneity among the mean number of differences in each of the four isolates. A *post hoc* Tukey’s HSD test was used to compare the means in the average and high isolates from the same population.

## Abbreviations

18S, 18S rRNA gene; 28S, 28S rRNA gene; bp, base pair; C_T_, threshold cycle; ETS, external transcribed spacer; EU, European; gm*Pok*1, gm*Pok*1 found outside rDNA; gm*Pok*2, gmPok2 found outside rDNA; g*Pokey*, *Pokey* elements found outside rDNA; g*Pokey*A, *Pokey*A found outside rDNA; g*Pokey*B, *Pokey*B found outside rDNA; IGS, intergenic spacer; ITS, internal transcribed spacer; kb, kilo base pairs; LTR, long terminal repeats; MITE, Miniature Inverted-repeat Transposable Element; NA, North American; PAE, percent amplification efficiency; qPCR, quantitative Polymerase Chain Reaction; rDNA, ribosomal DNA; rm*Pok*1, m*Pok*1 in the 28S gene; rm*Pok*2, m*Pok*2 in the 28S gene; r*Pokey*, *Pokey* elements found in the 28S gene; r*Pokey*A, *Pokey*A in the 28S rRNA gene; r*Pokey*B, *Pokey*B in the 28S rRNA gene; rRNA, ribosomal RNA; TE, transposable element; TIR, terminal inverted repeat; tm*Pok*1, m*Pok*1 in the genome; tm*Pok*2, m*Pok*2 in the genome; t*Pokey*, *Pokey* elements found in the genome; t*Pokey*A, *Pokey*A in the genome; t*Pokey*B, *Pokey*B in the genome; TSD, target site duplication.

## Additional files

Additional file 1: Table S1. Details of *Daphnia* isolates analyzed in this study.** Table S2.** C_T_ values from qPCR analysis of *Pokey*, m*Pok*, and rRNA gene number in 45 isolates from the *Daphnia pulex* complex.** Table S3.** Estimates of *Pokey*, m*Pok*, and rRNA gene number in 45 isolates of the *Daphnia pulex* complex. (XLSX 51 kb) Additional file 2: Figure S1. Correlation between 18S and 28S rRNA gene number *D. pulex* lineages. **Figure S2.** Correlation between 28S rRNA gene and r*Pokey* family number in *D. pulex* lineages. **Figure S3.** Repeat landscape for m*Pok* from *Daphnia arenata* isolate AR1.1. (PDF 34 kb) Additional file 3: Details of qPCR methods. (PDF 232 kb)
