# Supplementary material for: Distribution of the DNA transposon family, Pokey in the Daphnia pulex species complex
Source: Mob DNA. 2016 Jun 17;7:11. doi: 10.1186/s13100-016-0067-7 (PMC4912750; doi:10.1186/s13100-016-0067-7)
Supplement: Additional file 2: Figure S1. — Correlation between 18S and 28S rRNA gene number D. pulex lineages. Figure S2. Correlation between 28S rRNA gene and rPokey family number in D. pulex lineages. Figure S3. Repeat landscape for mPok from Daphnia arenata isolate AR1.1. (PDF 34 kb) [file 13100_2016_67_MOESM2_ESM.pdf]

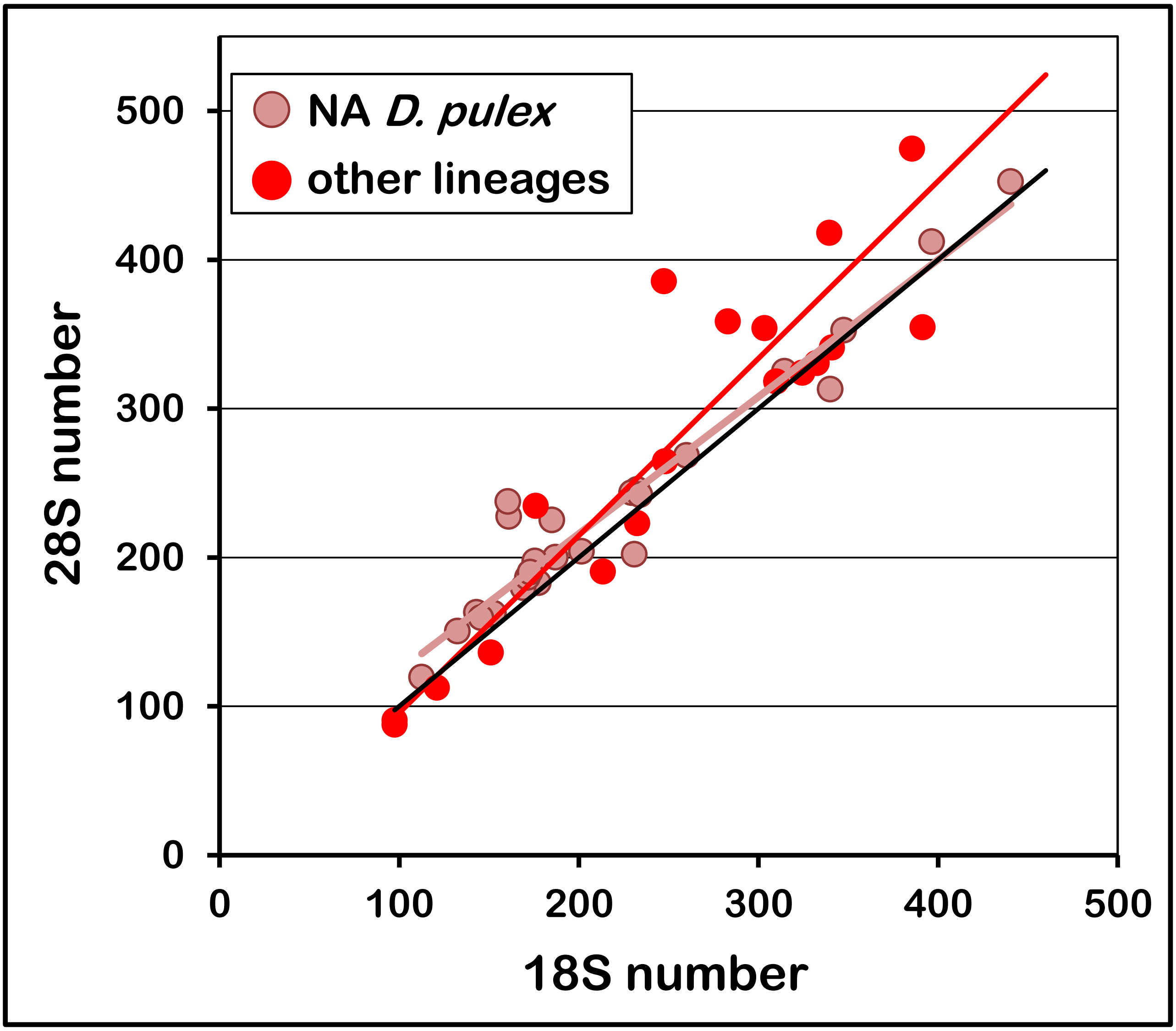

Figure S1. Correlation between 18S and 28S rRNA gene number *D. pulex* lineages.

There are two sets of isolates; (1) 26 isolates of North American *D. pulex* and (2) 19 isolates from four other lineages in the *D. pulex* complex. There is a significant positive correlation between 18S and 28S number in both sets of isolates (Table 2). The black line was generated by plotting the number of 18S genes in all isolates on both axes.

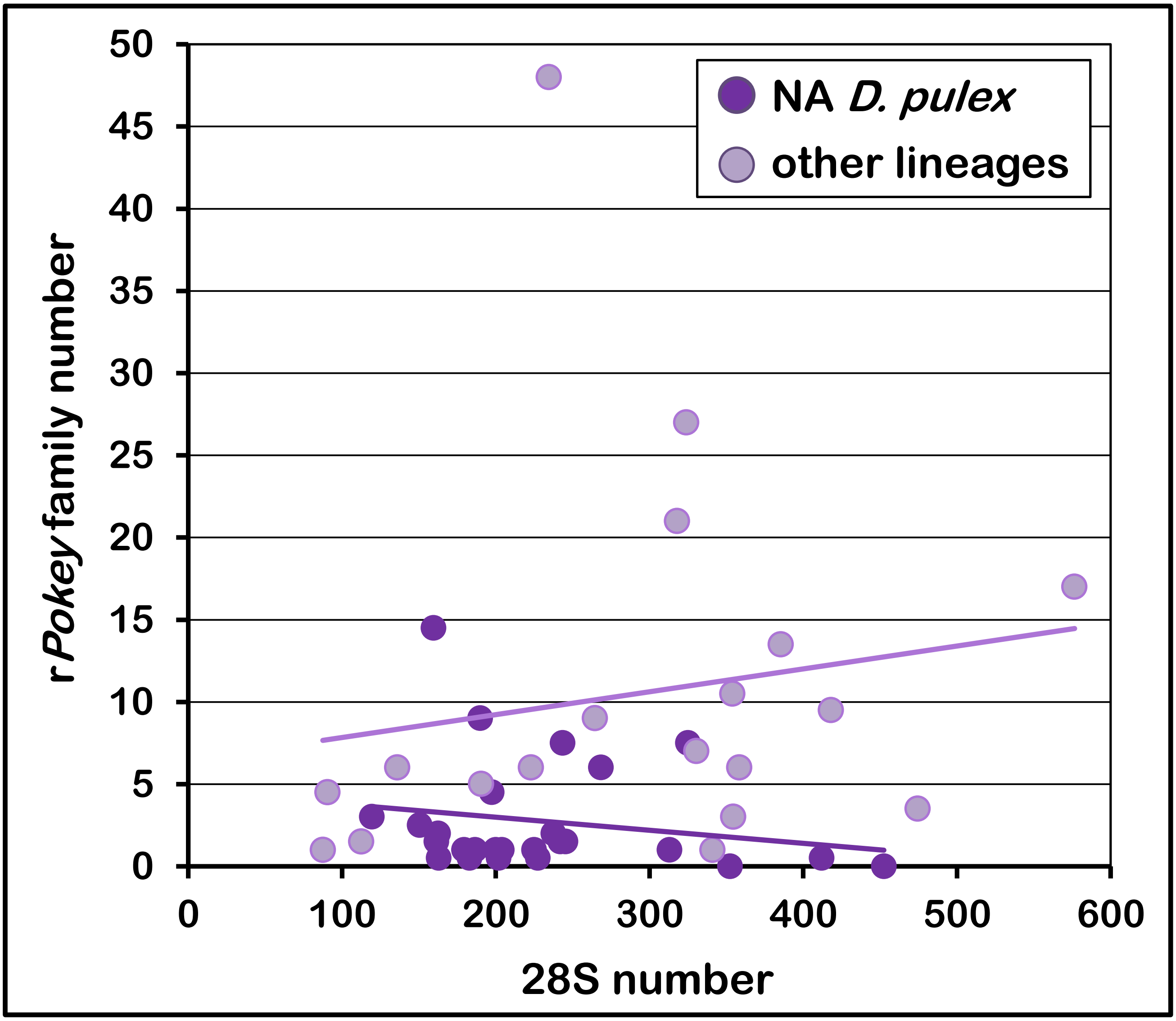

Figure S2. Correlation between 28S rRNA gene and r*Pokey* number in *D. pulex* lineages.
There are two sets of isolates; (1) 26 isolates of North American *D. pulex* and (2) 19 isolates from four other lineages in the *D. pulex* complex. No significant correlation was found between 28S and r*Pokey* family number (Table 2).

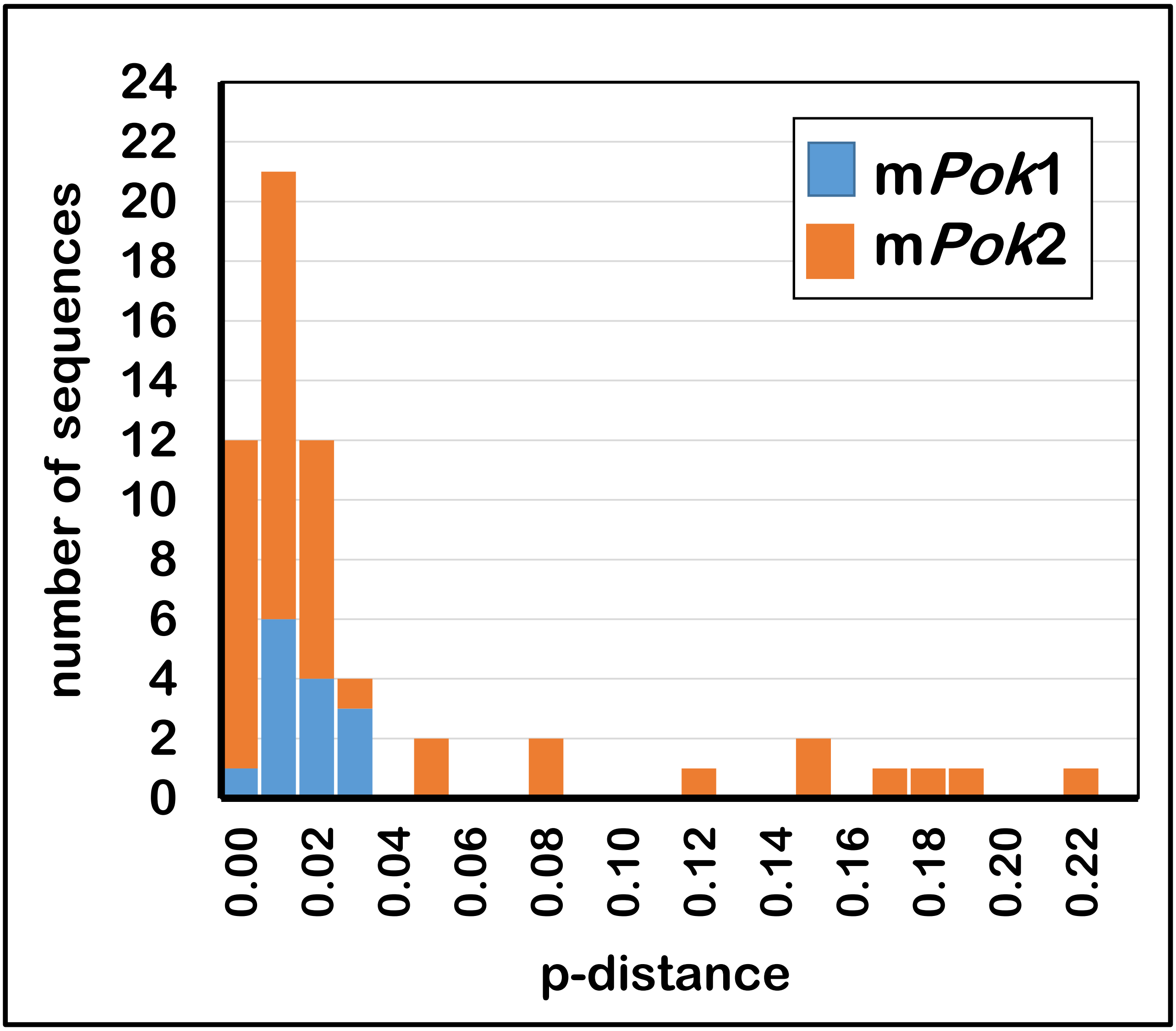

Figure S3. Repeat landscape for m*Pok* from *Daphnia arenata* isolate AR1.1.

This analysis is based on the m*Pok**1*** (14) and m*Pok*2 (46) sequences identified in the *Daphnia* genome sequence by Elliott et al. [6]. Uncorrected p-distance was calculated between the consensus of each group and each cloned sequence. Gaps were included in the analysis. Consecutive gaps were considered to be single nucleotide differences.
