# Supplementary material for: Distribution of the DNA transposon family, Pokey in the Daphnia pulex species complex
Source: Mob DNA. 2016 Jun 17;7:11. doi: 10.1186/s13100-016-0067-7 (PMC4912750; doi:10.1186/s13100-016-0067-7)
Supplement: Additional file 3: — Details of qPCR methods. (PDF 232 kb) [file 13100_2016_67_MOESM3_ESM.pdf]

Additional File 3: Additional qPCR methods

qPCR primers

qPCR primers were designed using Primer Express 2.0 (Applied Biosystems). The 18S rRNA gene (18S), *Tif*, and *Gtp* primer pairs (Table 4) were designed by McTaggart et al. (2007). *Tif* is a transcription initiation factor, *Gtp* is a member of the Rab superfamily of small GTPases, and the two genes were used as single-copy reference genes by McTaggart et al. (2007) and Eagle and Crease (2012). We designed one 28S rRNA gene (28S) primer pair and seven *Pokey* family primer pairs, t*Pokey*A, t*Pokey*B, tm*Pok*1, tm*Pok*2, r*Pokey*A+rm*Pok*1, r*Pokey*B, and rm*Pok*2 (Table 4). We used *Pokey* and m*Pok* sequences from the *Daphnia* genome (Colbourne et al., 2011) identified by Elliott et al. (2013) as reference sequences. *Pokey* and m*Pok* primer pair cross-reactivity was tested using a PCR with a final volume of 25 µL. The reactions contained 1X GenScript buffer (GenScript USA Inc.), 0.04 mM dNTPs, 0.04 µM of each primer, and 0.5 units of GenScript DNA polymerase (GenScript USA Inc.). Reactions were run using the following protocol: 2 min initial denaturation at 94ºC; 35 cycles of 30 sec denaturation at 94ºC, 30 sec annealing at 60ºC, and 1 min extension at 72ºC; followed by a final elongation at 72ºC for 5 min. The cross-reactivity tests involved using each t*Pokey* or m*Pok* primer pair (t*Pokey*A, t*Pokey*B, tm*Pok*1, tm*Pok*2) on each type of *Pokey* or m*Pok* template (*Pokey*A, *Pokey*B, m*Pok*1, rm*Pok*2) and each r*Pokey* or rm*Pok* primer pair (r*Pokey*A+rm*Pok*1, r*Pokey*B, rm*Pok*2) on each type of rDNA *Pokey* or m*Pok* template (r*Pokey*A, r*Pokey*B, rm*Pok*2).

Standard curves to estimate PAEs

Standard curves were run to estimate the Percent Amplification Efficiency (PAE) for each primer pair. Template DNA for the standard curve plates were amplicons (TableS3.1) generated from plasmid DNA (TableS3.2).

Cloning *Pokey* from isolate AR1.1

To generate plasmids with *Pokey*B, m*Pok*1, m*Pok*2 + the 28SrRNA gene downstream of the *Pokey* insertion site, and the 3' end of *Pokey*B + the 28S rRNA gene downstream of the *Pokey* insertion site the desired fragments were cloned from isolate

AR1.1, which is the isolate whose genome was sequenced (Colbourne et al., 2011). *Pokey*B, m*Pok*1, m*Pok*2 + 28S, and *Pokey*B + 28S were amplified in 25 µL reactions containing 1X Phusion HF or GC Buffer (NEB), 0.2 mM dNTPs, 0.2 µM of each primer, and 0.6 units of Phusion DNA Polymerase. Reactions were run as follows: 2 min initial denaturation at 94ºC; 35 cycles of 30 sec denaturation at 94ºC, 30 sec annealing at 60ºC, and 1 min extension at 72ºC; followed by a final elongation at 72ºC for 5 min. Extension conditions for the *Pokey*B clone were as follows: a 4 min extension for the first 20 cycles followed by a 4 min + 20sec/cycle extension for the last 10 cycles. Primers and deviations from these standard conditions are outlined in TableS3.4. The amplicons were cloned into the plasmid, pSC-B-amp/kan using the StrataClone Blunt PCR Cloning Kit (Agilent Technologies) as per the manufacturer's protocol with a few modifications. The modifications were as follows: 2-2.5 µL of PCR product and 0.5-1 µL of StrataClone Blunt Vector Mix were used in the ligation; 16-50 µL of StrataClone SoloPack cells and 0-32 µL of $CaCl_2$ were used for the transformation; an incubation of 30 minutes was used for large inserts; 500 µL of Terrific Broth was used; and 50 µL and 400 µL were plated. Clones were screened with colony PCR and DNA sequencing. Plasmid DNA was extracted from *Escherichia coli* (StrataClone Solopack Competent Cells) using the Roche High Pure plasmid isolation kit (Roche Applied Science), as per manufacturer's directions with a few modifications. The modifications were as follows: plasmid DNA was eluted in H_2O and the elution volume was cut in half.

Generating amplicons

Amplicons (TableS3.1) were generated using 1 to 30 ng of plasmid DNA in 25 µL PCRs containing 1X GenScript buffer (GenScript USA Inc.), 0.04 mM dNTPs, 0.05 µM of each primer, and 0.5 units of GenScript DNA polymerase (GenScript USA Inc.). Reactions were run using the following protocol: 2 min initial denaturation at 94ºC; 35 cycles of 30 sec denaturation at 94ºC, 30 sec annealing at 55ºC, and 90 sec extension at 72ºC; followed by a final elongation at 72ºC for 5 min. When generating the *Pokey*B amplicons, a 2 min extension was used. Five PCRs were run and pooled for each amplicon type. The E.Z.N.A. Micro-Spin DNA Clean-Up Kit (Omega Bio-Tek) was used to remove excess reagents from the amplicons, as per the manufacturer's protocol.

The concentration of the pooled amplicons was measured using a NanoDrop® ND-8000 spectrophotometer (ThermoScientific). The pooled amplicons were used to generate serial dilutions for the standard curve plates as in Eagle and Crease [2012].

Estimating PAE

PAEs were estimated using the procedure described in Eagle and Crease [2012], which involved generating standard curves for each primer pair, and using the slope of the line of best fit to calculate PAE = ln [10^(-1/slope)] / ln 2. As in Eagle and Crease [2012], templates for the standard curve plates were amplicons (TableS3.1) generated from plasmid DNA (TableS3.2). Six standard curve plates were run. The single copy gene primer pairs were included on each plate along with various combinations of four or five of the ten multicopy gene primer pairs. This produced three PAE estimates for each multicopy gene primer pair and six PAE estimates for the two single copy gene primer pairs (TableS3.4, FigureS3.1). For each primer pair, the standard deviation was calculated from the three (or six) PAE values, and this was used to estimate the 95% confidence interval, assuming a normal distribution (Microsoft Excel, Richmond, Washington, USA). If an individual PAE value fell outside the 95% confidence interval for its primer pair, then that estimate was omitted, and the mean PAE for that primer pair was recalculated. The recalculated mean PAE values were used in the calculation of gene numbers to correct for differences in primer efficiency.

Screening for rm*Pok*1

Because the r*Pokey*A + rm*Pok*1 primer pair amplified both r*Pokey*A and rm*Pok*1 , we wanted to verify whether the genes amplified with this primer pair were *Pokey*A, m*Pok*1, or a combination of the two. If there were no t*Pokey*A, then in theory there are no r*Pokey*A . In addition, if there were not tm*Pok*1, then in theory there are no rm*Pok*1. Most isolates only had t*Pokey*A suggesting that the genes amplified with the r*Pokey*A + rm*Pok*1 primer pair were r*Pokey*A. However, one isolate AR3.1 contained both t*Pokey*A and tm*Pok*1. Therefore, we used end-point PCR to verify whether this isolate contained only r*Pokey*A, only rm*Pok*1, or both. Reactions had a final volume of 25 µL and contained 1X GenScript buffer (GenScript USA Inc.), 0.04 mM dNTPs, 0.04 µM of

each primer, and 0.5 units of GenScript DNA polymerase (GenScript USA Inc.). Reactions were run using the following protocol: 2 min initial denaturation at 94ºC; 35 cycles of 30 sec denaturation at 94ºC, 30 sec annealing at 55ºC, and 1 min extension at 72ºC; followed by a final elongation at 72ºC for 5 min. The mPok1-530F (5' cta tgg cgg gat ttt tcg c) and 28S-3073R (5' tga cga ggc att tgg cta cc) primers were used.

References

Colbourne JK, Pfrender ME, Gilbert DG, Thomas WK, Tucker A, Oakley TH, et al. The ecoresponsive genome of *Daphnia pulex*. Science. 2011;331:555–61.

Crease, T.J. & Colbourne, J.K. 1998. The unusually long small-subunit ribosomal RNA of the crustacean, *Daphnia pulex*: sequence and predicted secondary structure. *Journal of Molecular Evolution* **46**: 307–313.

Eagle SH, Crease TJ. Copy number variation of ribosomal DNA and *Pokey* transposons in natural populations of *Daphnia.* Mobile DNA. 2012;3:4.

Elliott T A, Stage DE, Crease TJ, Eickbush TH. In and out of the rRNA genes: characterization of *Pokey* elements in the sequenced *Daphnia* genome. Mobile DNA. 2013;4:20.

McTaggart SJ, Dudycha JL, Omilian A, Crease TJ. Rates of recombination in the ribosomal DNA of apomictically propagated *Daphnia obtusa* lines. Genetics. 2007;175:311–20.

Omilian AR, Lynch M. Patterns of intraspecific DNA variation in the *Daphnia* nuclear genome. Genetics. 2009;182:325–36.

Penton EH, Crease TJ. Evolution of the transposable element *Pokey* in the ribosomal DNA of species in the subgenus *Daphnia* (Crustacea: Cladocera). Mol Biol Evol. 2004;21:1727–39.

TableS3.1. Amplicons used as templates to generate the qPCR standard curve.

PCR amplicon name	qPCR primer set	Template DNA (Plasmid Name)	Primers	Primer Sequence (5'-3')	Amplicon size (bp)
18S	18S	18S	18S 1522 F<br>18S 2004 R	att ccg ata acg aac gag<br>tgg gga tca ttg cag tcc cca atc	482
28S	28S	28S	28S 2292 F<br>28S 3204 R	acg cga cac aaa ccg gag aag<br>gag tca agc tca aca ggg tct tct ttc cc	912
Tif	*Tif*	*Tif*	M13 F<br>M13 R	gtt gta aaa cga cgg cca gtg<br>cag gaa aca gct atg acc atg	730
Gtp	*Gtp*	*Gtp*	M13 F<br>M13 R	gtt gta aaa cga cgg cca gtg<br>cag gaa aca gct atg acc atg	707
*Pokey*A	t*Pokey*A	Pok 330F & 5394R	Pok 2904 F<br>Pok 3811 R	ctt cga ggc gct att agt gc<br>ccg tgt tac ttc acc atc gg	907
*Pokey*B	t*Pokey*B	PokeyB-4	Pok 2904 F<br>Pok 5128 R	ctt cga ggc gct att agt gc<br>cga ccc gaa acg cct tag t	2128
m*Pok*1	tm*Pok*1	mPok1-16	M13 F<br>M13 R	gtt gta aaa cga cgg cca gtg<br>cag gaa aca gct atg acc atg	1020
rm*Pok*2	tm*Pok*2, rm*Pok*2	rmPok2-1	M13 F<br>M13 R	gtt gta aaa cga cgg cca gtg<br>cag gaa aca gct atg acc atg	531
r*Pokey*A	r*Pokey*A + rm*Pok*1	Pokey-28S	Pok 6172 F<br>28S 3104 R	tgg tcg atg gta aag acc tca acg tc<br>gtt aat cca ttc gtg cgc g	486
r*Pokey*B	r*Pokey*B	rPokeyB-7	M13 F<br>M13 R	gtt gta aaa cga cgg cca gtg<br>cag gaa aca gct atg acc atg	515

bp = base pair, 18S = 18S rRNA genes, 28S = 28S rRNA genes, t*Pokey* = total *Pokey*, tm*Pok* = total m*Pok*, r*Pokey* = *Pokey* inserted in 28S, rm*Pok* = m*Pok* inserted in 28S.

TableS3.2. Plasmid DNA used for generation of amplicons used as templates for the qPCR standard curves.

PCR amplicon name	Plasmid Clone	Insert Description	Insert size (bp)	Vector	Vector size	Total plasmid size (bp)	Reference
18S	18S	3.7 kb insert with entire 18S rRNA gene	3789	pBluescript-KS+	2961	6750	Colbourne and Crease, 1998
28S	28S	3.4 kb 28S gene insert	2596	pSC-B-amp/kan	4272	6868	Eagle and Crease, 2012
Tif	*Tif*	partial *Tif* gene	561	pCR4-TOPO	3956	4517	Omilian and Lynch, 2009
Gtp	*Gtp*	partial *Gtp* gene	538	pCR4-TOPO	3956	4494	Omilian and Lynch, 2009
*Pokey*A	Pok330F& 5394R	5064 bp of internal region of *Pokey*	5064	pCR4-TOPO	3956	9020	Crease, unpublished
*Pokey*B	PokeyB-4	full *Pokey*B with 76 bp of 28S gene at 5'end	4469	pSC-B-amp/kan	4272	8741	current study
m*Pok*1	mPok1-16	full m*Pok*1	746	pSC-B-amp/kan	4272	5018	current study
rm*Pok*2	rmPok2-1	204 bp for m*Pok*2 3' end and 53 bp of 28S gene	257	pSC-B-amp/kan	4272	4529	current study
r*Pokey*A	Pokey-28S	~1500 bp of *Pokey* 3' end and ~200 nt of 28S gene	1726	pCR4-TOPO	3956	5682	Penton and Crease, 2004
r*Pokey*B	rPokeyB-7	188 bp of *Pokey*B 3' end and 53 bp of 28S gene	241	pSC-B-amp/kan	4272	4513	current study

bp = base pairs, 18S = 18S rRNA genes, 28S = 28S rRNA genes, r*Pokey* = *Pokey* inserted in 28S, rm*Pok* = m*Pok* inserted in 28S.

TableS3.3- Amplification details for *Pokey* elements from isolate AR1.1.

Final Plasmid Name	Insert Description	Primer<br>F = forward<br>R = reverse	Extra $MgCl_2$ (mM)	dNTP (mM)	Primers (μM)	DNA Pol (units)
**mPok1-16**	full mPok1	mPok1-3F<br>5’ aa ccc ttt ttc gac tga cgg<br><br>mPok1-750R<br>5’ aa ccc ttt atc gac cgc cac				
**PokeyB-4**	full *Pokey*B with 76 bp of 28S gene at 5'end	28S-2974F<br>5’ ctg ccc agt gct ctg aat gtc aaa gtg aag<br><br>PokB-4330R<br>5’ aac cct ttt tcg acg cca aag	0.25	0.4	0.05	
**rmPok2-1**	204 bp for m*Pok*2 3' end and 53 bp of 28S gene	mPok2-556F<br>5’ ggg aca tag gtg tcc cgg<br><br>28S-3104R<br>5’ gtt aat cca ttc gtg cgc g	0.25	0.1		0.2
**rPokeyB-7**	188 bp of *Pokey*B 3' end and 53 bp of 28S gene	PokB-4151F<br>5’ ttt ttc ggt gac tga agt gcc<br><br>28S-3104R<br>5’ gtt aat cca ttc gtg cgc g	0.25	0.1		0.2

The PCR details are shown only if they deviate from general conditions. DNA Pol = DNA polymerase, Extra $MgCl_2$ = the amount of $MgCl_2$ used in addition to the 1.5 mM supplied in the buffer, and Temp Anneal = annealing temperature.

TableS3.4. PAE estimates from each of the six standard curve plates.

Primer Set	PAE1	PAE2	PAE3	PAE4	PAE5	PAE6	Mean	Std dev	95% conf	95% low	95% high	Recal Mean
18S		0.949		0.945	~~0.971~~		0.955	0.014	0.016	0.939	0.971	0.947
28S		0.943		0.937		0.932	0.937	0.005	0.006	0.931	0.944	0.937
t*Pokey*A	0.868			~~0.862~~		0.868	0.866	0.004	0.004	0.862	0.870	0.868
t*Pokey*B	0.898			0.882	0.911		0.897	0.015	0.017	0.880	0.914	0.897
tm*Pok*1	~~0.844~~		0.875		0.874		0.864	0.017	0.020	0.845	0.884	0.874
tm*Pok*2	~~0.884~~		0.909		0.913		0.902	0.016	0.018	0.884	0.920	0.911
r*Pokey*A+rm*Pok*1	0.904		0.923			0.915	0.914	0.010	0.011	0.903	0.925	0.914
r*Pokey*B		0.900	0.895		0.914		0.903	0.010	0.011	0.892	0.914	0.903
rm*Pok*2		0.900	0.903			0.898	0.900	0.003	0.003	0.897	0.904	0.900
Tif	0.935	0.937	~~0.956~~	~~0.921~~	~~0.951~~	0.936	0.939	0.013	0.010	0.929	0.949	0.936
Gtp	0.926	0.923	0.931	~~0.914~~	~~0.942~~	0.934	0.928	0.010	0.008	0.920	0.936	0.928

Six standard curve plates were run, generating three PAE estimates for each multicopy gene primer pair and six PAE estimates for the two single copy gene primer pairs (PAE1-6). For each primer pair, the standard deviation (Std dev) was calculated from the three (or six) PAE values, and this was used to estimate the 95% confidence interval (95% conf). If an individual PAE value fell outside the 95% confidence interval (95% low – 95% high) for its primer pair, that estimate was omitted (indicated with a strikethrough), and the mean PAE for that primer pair was recalculated (Recal Mean). 18S = 18S rRNA genes, 28S = 28S rRNA genes, t*Pokey* = total *Pokey*, tm*Pok* = total m*Pok*, r*Pokey* = *Pokey* inserted in 28S, rm*Pok* = m*Pok* inserted in 28S.

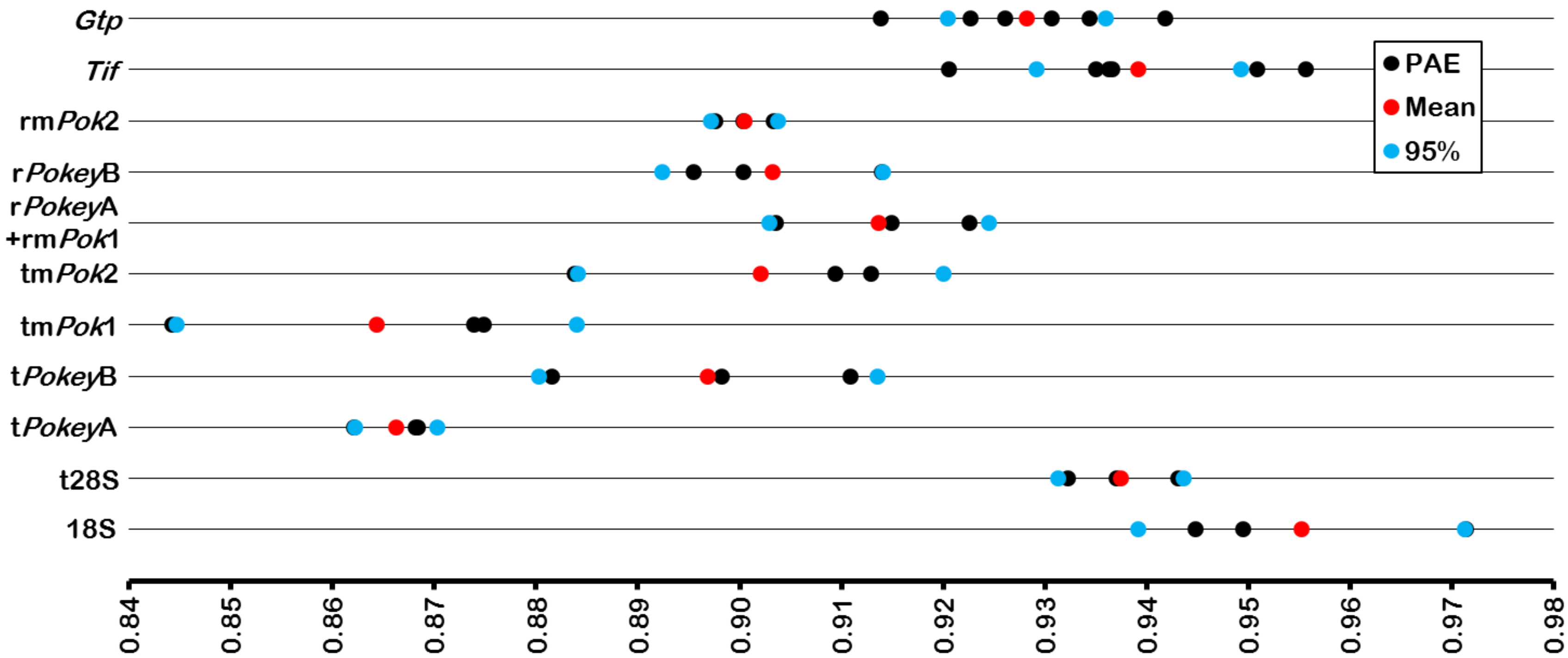

FigureS3.1. Graphical representation of data from Table.
Blue (95%) = lower and upper end of 95% confidence interval. 18S = 18S rRNA genes, 28S = 28S rRNA genes, *Pokey* = total *Pokey*, tm*Pok* = total m*Pok*, r*Pokey* = *Pokey* inserted in 28S, rm*Pok* = m*Pok* inserted in 28S.
